# Fracture Resistance of Endodontically Treated Premolars Reconstructed by Traditional Casting and CAD-CAM Milling Post and Cores

**DOI:** 10.1155/2022/6736303

**Published:** 2022-09-22

**Authors:** Davood Nodehi, Azizollah Moraditalab, Shahrzad Shafiee, Salehe Sekandari, Farzaneh Ahrari

**Affiliations:** ^1^Dental Research Center, Department of Prosthodontics, School of Dentistry, Mashhad University of Medical Sciences, Mashhad, Iran; ^2^Dentist, Mashhad, Iran; ^3^Dental Research Center, Department of Cosmetic and Restorative Dentistry, School of Dentistry, Mashhad University of Medical Sciences, Mashhad, Iran; ^4^Dental Research Center, School of Dentistry, Mashhad University of Medical Sciences, Mashhad, Iran

## Abstract

**Purpose:**

Restoration of endodontically treated premolars has always been considered as a challenging procedure. This study compared the fracture strength and mode of failure of root canal treated premolars reconstructed with various post and core systems.

**Materials and Methods:**

Twenty healthy extracted premolars were selected and underwent root canal treatment and then randomly assigned into 4 groups (*n* = 5). The teeth in group 1 restored with amalgam, whereas others reconstructed with post and cores made by cobalt-chromium (Co-Cr) casting (group 2), nonprecious gold (NPG) casting (group 3), or computer-aided design (CAD) and computer-aided manufacturing (CAM) milling (group 4). The force at fracture was measured in a universal testing machine, and the failure mode was recorded as repairable or nonrepairable.

**Results:**

ANOVA revealed a significant difference in fracture resistance between groups (*P*=0.001). The control group displayed significantly lower strength than that of the CAD-CAM or CO-Cr groups (*P* < 0.05). The CAD-CAM posts were also more resistant to fracture than the NPG group (*P* < 0.05). The frequencies of repairable fracture in the control, Co-Cr, NPG, and CAD-CAM groups were 40%, 20%, 20%, and 60%, respectively. The chi-square test revealed no significant difference in the distribution of failure modes between groups (*P*=0.415).

**Conclusion:**

The teeth reconstructed with post and cores were more resistant to fracture than those restored with amalgam alone. CAD-CAM milling could be considered as the best system for reconstruction of endodontically treated teeth, as it provided the highest fracture strength with less risk of nonrepairable tooth fracture.

## 1. Introduction

Pulpless teeth generally display a higher fracture rate than vital teeth [1, 2]. Traditionally, it was believed that endodontic treatment makes the teeth fragile due to dehydration and impairment of the nerve reaction mechanism [3]. Recent findings indicated that despite the moisture reduction by 9% in endodontically treated teeth, the mechanical characteristics of dentin in terms of strength and hardness are not much different from the dentin of intact teeth [4, 5]. Today, the main determinant for selecting the type of restoration for an endodontically treated tooth is the amount of residual tooth structure [6, 7]. In cases with sufficient dentin, it is better to supply retention from the pulp chamber, but if a great extent of dentin has been lost as a result of extensive caries, previous restorations, or access cavity preparation, the application of posts for enhancing the internal strength of the teeth is inevitable [8]. This is especially true for premolars, as these teeth usually have insufficient dentin after endodontic therapy and thus require root canal retention for the long-term maintenance of restorations and minimizing the possibility of fracture [9, 10].

Different types of posts are available for enhancement of endodontically treated teeth such as cast posts, prefabricated metal posts, and fiber-reinforced composite posts. Choosing an appropriate type of post and core among many of them could be a challenging issue in the clinical conditions. Historically, the cast metal post and cores are considered as the standard choice for reconstruction of endodontically treated teeth. However, they are rigid and have high modulus of elasticity, which may cause severe fractures [11–13]. The cast posts are made from various metal alloys including cobalt-chromium (Co-Cr), nickel-chromium (Ni-Cr), gold, or nonprecious gold (NPG), which are different in hardness, expense, and strength [14]. The NPG alloy was introduced in 1987 and contains more than 80% copper. It displays suitable physical properties such as easy casting, excellent fit, optimal polishability, and favorable biocompatibility [15]. Furthermore, NPG provides high durability and strength, and its modulus of elasticity is lower than that of Co-Cr or Ni-Cr castings (Table 1). Recently, computer-aided design (CAD) and computer-aided manufacturing (CAM) technology has been employed for fabrication of a variety of prostheses, such as post and cores [16]. In this way, the frameworks made from the Co-Cr alloy are reduced by CAD-CAM milling (Table 1). The use of CAD-CAM system for post-manufacturing is associated with multiple benefits such as reducing the construction cost and material usage, saving time required for the design and construction of the posts, eliminating the manufacturing and human errors, and enhancing the accuracy of fitting [19–23].

There are conflicting opinions about the effect of post and core systems on the resistance to fracture of root canal treated teeth. Some studies indicated that placing the post in the canal would increase the stress tolerance by the tooth and thus reducing the risk of fracture [24, 25]. Other studies found a rise on the risk of fracture following placement of posts in the root canals [26, 27]. The effect of different post systems on the survival rate of the teeth is also a matter of controversy between studies. Furthermore, the literature contains little information about the effectiveness of posts and cores made from the NPG alloy, or those fabricated by CAD-CAM milling technique when applied for rebuilding of premolar teeth. Therefore, the present study was conducted to compare the effect of different post and core systems (Co-Cr casting, NPG casting, and CAD-CAM milling) on the fracture toughness and mode of failure of root canal treated premolars. The hypothesis of this study was that due to the lower modulus of elasticity of CAD-CAM and NPG posts, the force would distribute better along the post and their performance would be higher than the conventional cast posts.

## 2. Materials and Methods

### 2.1. Sample Preparation

In this experimental study, twenty intact, single-rooted mandibular premolars were collected, cleaned, and stored in 0.9% saline solution until the time of the experiment. The selected teeth were healthy and without caries, fracture, cervical abrasion, or restoration. The teeth underwent root canal therapy by the step-back technique, and the canals were obturated through lateral condensation using 40-size gutta-percha as a master cone.

### 2.2. The Procedures Performed in the Study Groups

#### 2.2.1. Control Group

Group 1: the 5 teeth in the control group were randomly selected from the total sample after endodontic treatment. In these teeth, the access cavity and the remaining crown were filled with amalgam (SDI, Bayswater, Victoria, Australia) without the use of root canal for retention.

#### 2.2.2. Experimental Groups

The remaining teeth were decoronated at 3 mm above the cementoenamel junction (CEJ) using a diamond fissure bur at a high-speed hand-piece with air and water spray. The root length as well as the mesiodistal and buccolingual dimensions of the teeth at 3 mm above the CEJ were measured with a digital caliper, and teeth with root length <15 mm after decoronation were excluded from the sample. Excluded were also the teeth with crown length and dimensions that deviated more than 1 mm from the mean values. The root canal orifices were filled with Cavit temporary restorative material (CAVISOL, Golchai Co, Tehran, Iran), and the specimens were kept in water at 37°C for 2 days. After that, Cavit was removed and the post space was prepared to the length of 10 mm, using Peeso reamer drills # 1 and 2 (Mani Inc, Tochigi, Japan). The prepared canal was washed with water and dried with paper cones (Meta Biomed Co, Seoul, Korea). The 15 treated teeth were then randomly assigned into three groups (groups 2 to 4; *n* = 5) and underwent treatment with various post and core techniques. Group 2 (Co-Cr casting): in this group, the impression was made with C-type heavy and light body silicon materials (Speedex, Coltene, Altstatten, Switzerland). The post and core pattern was then formed with inlay wax on the dental stone model (Neo-Stone Din EN ISO6873, Type 4, Siladent, Germany) and fabricated by the casting technique using the Co-Cr alloy (Aalba Dent, Inc, Fairfield, CA, USA). After post fabrication, the canal was cleaned with 5% sodium hypochlorite (NaOCl), thoroughly rinsed with water, and dried with paper cones. The post and core were also cleaned with 70% ethanol and then cemented by the glass ionomer cement (GC Co, Tokyo, Japan). A firm pressure was used for positioning the post in place, and the excess material was removed.

Group 3 (NPG casting): the procedure was the same as that described in group 2, but the NPG alloy (Aalba Dent Inc Fairfield, CA, USA) was used in the casting process.

**Figure 1:**
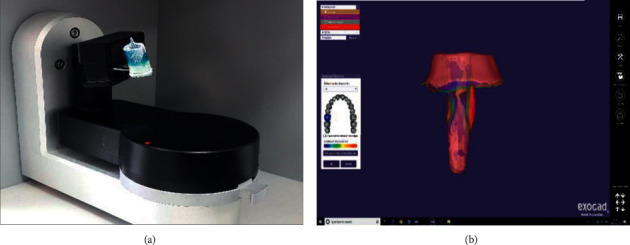
Scanning (a) and digital design (b) of a CAD-CAM milling post and core.

Group 4 (CAD-CAM milling): in this group, the impression procedure was performed similar to groups 2 and 3. Then, a digital cast was made instead of a physical cast, through scanning of the impression with a digital scanner (desktop scanner Iscan D104i max. resolution 0.005 mm, Imetric 4D Imaging Sàrl, Courgenay, Switzerland). The post and core were designed through special software using the three-dimensional cast images. The standard template library (STL) files were prepared and transferred to the milling machine (Ceramill Matik, Amann Girrbach AG, Koblach, Austria) for fabricating the post and core from nickel- and beryllium-free Co-Cr-Mo metal blocks (Ceramill Sintron, Amann Girrbach, Germany). After milling, the post underwent thermal processing in an argon atmosphere at high temperature. Heat treatment caused about 11% shrinkage, which was considered by the technician during the designing process (Figure 1). The posts were then cemented, as explained in group 2.

### 2.3. Measuring the Fracture Strength

The restored teeth were kept in distilled water at room temperature for 7 days. After that, the teeth were mounted vertically at the level of CEJ in clear acrylic resin (Acropars, Iran) to allow visualization of the fracture site. The mounted specimens were placed in a universal testing machine (SANTAM Co, Iran) with a 500 N load cell. A compressive load was applied at an angle of 135° relative to the long axis of the tooth (45° relative to the horizontal plane). The load was exerted 2 mm below the occlusal edge of the core at a crosshead speed of 0.5 mm/min, and the force at fracture was recorded in Newton (N) and considered as the fracture resistance value.

### 2.4. Type of Failure

After measuring the fracture strength, the specimens were examined at 10 X magnification to determine the type of failure. The fracture modes were categorized as repairable or catastrophic (nonrepairable). If the site of fracture was at the level or above the acrylic resin, it was considered repairable; otherwise, the fracture type was considered catastrophic. The catastrophic category included both irreversible horizontal fracture and vertical root fracture.

### 2.5. Statistical Analysis

The normality of the data distribution was confirmed by the Shapiro–Wilk test (*P* > 0.05). One-way analysis of variance (ANOVA) was run to detect any significant difference in fracture resistance among the study groups, followed by Tukey post hoc test for pairwise comparisons. The difference in the distribution of fracture types was assessed by the chi-square test. The data were analyzed through SPPS software (version 16.0; SPSS In, Chicago, Il), and a *P*-value <0.05 was considered statistically significant.

## 3. Results

### 3.1. Comparison of Fracture Resistance between Groups

Table 2 shows the mean and standard deviation (SD) of fracture resistance (N) of restored teeth in the study groups. The lowest mean fracture strength belonged to the control group (6.00 ± 0.42 N) and the highest was observed in the CAD-CAM group (7.09 ± 0.12 N).

ANOVA revealed a statistically significant difference in fracture strength between the four groups (*P*=0.001, Table 2). Pairwise comparisons showed that the mean fracture resistance in the control group was significantly lower than that of the CAD-CAM and CO-Cr groups (*P* < 0.05; Table 2). Furthermore, the posts in the NPG group exhibited significantly lower strength than that of the CAD-CAM group (*P* < 0.05; Table 2). The difference in fracture resistance values between the other groups was not statistically significant (*P* > 0.05).

### 3.2. Comparison of Failure Modes between the Study Groups

**Figure 2:**
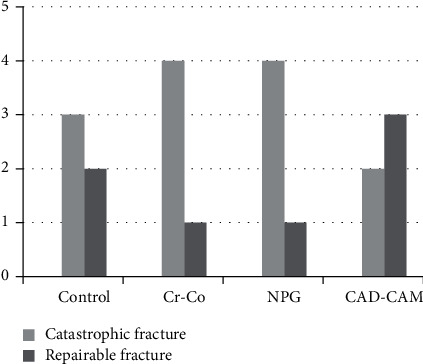
Distribution of failure types in the study groups.

The distribution of failure modes is illustrated in Figure 2. Repairable and catastrophic fractures were observed in 35% and 65% of the samples, respectively. Only one case of vertical root fracture occurred among the specimens. The frequencies of repairable fracture in the control, Co-Cr, NPG, and CAD-CAM groups were 40%, 20%, 20%, and 60%, respectively. Catastrophic fractures were found in 3 of the 5 specimens in the control group (60%), 4 from 5 (80%) in both the Co-Cr and NPG cast groups, and 2 (one horizontal and one vertical root fractures) from 5 (40%) in the CAD-CAM milling group. Fisher's exact test revealed no significant difference in the fracture mode distribution among the study groups (*P*=0.742).

## 4. Discussion

The present in vitro study investigated the fracture strength and mode of failure of endodontically treated premolars reconstructed with different post and core systems (Co-Cr casting, NPG casting, and CAD-CAM milling). Fracture resistance of root canal-filled teeth depends primarily on the remaining dentin structure [28, 29]. Therefore, teeth with relatively similar lengths and dimensions were included in the present study. The specimens were tested without placing full crowns, and the load was exerted directly on the cores. The use of full coverage restorations during evaluating fracture strengths of root canal treated teeth has been questioned, as the crown placed over a core buildup could create a ferrule effect when its margins encircle a sound dentin collar, that enhances fracture resistance of the restoration [30, 31].

The present findings indicated that the premolar teeth reconstructed with posts and cores were more resistant to fracture than those restored with amalgam alone. Furthermore, the CAD-CAM milling post and core technique provided the highest fracture resistance, after that was the Co-Cr cast group. According to the statistical analysis, Co-Cr posts made by traditional casting or CAD/CAM milling displayed comparable fracture resistance, which was significantly greater than that of the control group. The NPG casting technique displayed lower performance than the other systems, as the fracture resistance of teeth reconstructed by NPG post and cores was significantly lower than that of the CAD-CAM milling group and comparable to both the control and Co-Cr groups. Therefore, the hypothesis of this study was rejected because the lower elastic modulus of NPG and CAD-CAM milling posts did not lead to enhanced fracture resistance of premolars, as compared to conventional Co-Cr posts.

The higher fracture load of CAD/CAM milling posts may be attributed to the more homogenous structure of metal and greater accuracy in the manufacturing process [21, 32, 33], whereas casting posts probably have more porosity in their structure. Furthermore, the alloy composition may influence the resistance to fracture. It is generally assumed that a more similar modulus of elasticity between the post and dentin would lead to a better force distribution along the length of the post and thus higher fracture load [34]. However, a review of literature by Creugers et al. [35] revealed a large variation in the survival rate of root canal treated teeth reconstructed with different post and core systems. Despite the lower modulus of elasticity of NPG and CAD-CAM milling posts in the present study, the difference in fracture resistance between these systems and Co-Cr posts was not statistically significant.

The outcomes of the present study are consistent with several studies that confirmed the benefits of post and core systems for increasing the stress tolerance of root canal treated teeth [13, 22, 24, 36, 37]. The present findings also corroborate the results of Bilgin et al. [14] who demonstrated the highest fracture resistance values in posts fabricated by computer-aided design and manufacturing (CAD-/CAM) milling. In contrast to the outcomes of this study, several studies [37–39] demonstrated a significant superiority of NPG posts as compared to the Co-Cr or Ni-Cr posts for reinforcing the tooth structure. This discrepancy between the results of this study and those of previous authors may be related to the differences in the method of post construction, or the use of full-coverage metal crowns instead of cores for force application. Furthermore, in some studies [37–39], the samples were embedded in a thin layer of silicone impression material or molten wax to provide space between the root and acrylic resin and thus partly mimic the response of periodontal ligament to natural loads, but this procedure was not performed in the present investigation.

Root fracture is considered as the etiologic factor for 3 to 10 percent of failures in teeth reconstructed with post and core systems [40]. Haghighi et al. [37] exhibited that core fracture was more predominant in nonvital teeth restored with amalgam alone, whereas teeth rebuilded with Ni-Cr or NPG posts displayed a high frequency of root fracture. Some studies suggested that a post with high modulus of elasticity is unable to absorb the shock, and thus localized points of stress are created within the root, which can lead to catastrophic fracture [41, 42]. This hypothesis was not confirmed in the present study because the difference in the type of failure was not significant between the study groups. However, most fractures in the NPG and Co-Cr groups were catastrophic; whereas the CAD-CAM posts showed the highest frequency of restorable failures. The frequency of vertical root fracture was rare, and most specimens showed horizontal tooth fracture in the present study.

The limitations of this study were the small sample size and the lack of exposing the specimens to the cyclic loading or thermal cycles to simulate the clinical conditions. Further studies with a larger sample and better simulation of the oral environments are warranted to compare the effectiveness of various post and core systems in root canal treated teeth.

## 5. Conclusion

Within the limitations of this in vitro study,

1. The teeth reconstructed with post and cores were more resistant to fracture than those restored with amalgam alone, and CAD-CAM milling post and cores provided the highest fracture resistance among the study groups.
2. The difference in the mode of fracture was not significant among the study groups, but the CAD-CAM system displayed the highest frequency of restorable failure mode.
3. CAD-CAM milling could be considered as the best system for reconstruction of endodontically treated teeth, as it provided the highest fracture strength with less risk of catastrophic tooth fracture.

## Figures and Tables

**Table 1:** Properties of alloys for fabricating the post and core in the study group.

Type	Ultimate tensile strength (MPa)	Yield strength at 0.2% offset (MPa)	Elongation (%)	VHN(kg/mm^2^)	Density (g/cm^3^)	Composition
Co-Cr [16]	755	675	6	359	8.7	Co 63.5% Cr 27.0% Mo 5.5% Fe 2.0% Ni, Si, Mn<1%
NPG [17]	560	265	15	140	7.8	Cu 80.7% Al 7.8% Ni 4.3% C, Si, Nb, Mn, Fe <1%
Co-Cr-Mo metal blocks [18]	597	413	12	288	8.3	Co 65% Cr 28% Mo 5% C, Si, Nb, Mn, Fe <1%

**Table 2:** Mean, standard deviation (SD), minimum (Min), and maximum (Max) values of fracture resistance (N) in the study group.

Group	Mean^*∗*^	SD	Min (*N*)	Max (*N*)	*P*-value (ANOVA)
Control	6.00^a^	0.42	5.68	6.73	F = 10.01 *P*=0.001
Co-Cr	6.66 ^bc^	0.31	6.35	7.08	
NPG	6.41^ab^	0.36	6.03	6.81	
CAD-CAM	7.09^c^	0.12	6.94	7.26	

^*∗*^Tukey pairwise comparison test; the groups that have been marked by different letters showed significant differences at *P* < 0.05.

## Data Availability

The data used to support the findings of this study are available from the corresponding author upon request.

## References

[1] Abyari S., Amini P., Amini R., Zafari A., Bami L. (2020). Evaluation of shears strength of dowel amalgam and post-amalgam in root canal-treated teeth. *Journal of Dental Materials and Techniques*.

[2] Jamshidy L., Parvaz A. (2020). Tensile strength of non-precious gold, cobalt-chromium, and fiber posts cemented with panavia *F*2 resin cement in root canals of endodontically-treated teeth. *Pakistan Journal of Medical and Health Sciences*.

[3] Makade C. S., Meshram G. K., Warhadpande M., Patil P. G. (2011). A comparative evaluation of fracture resistance of endodontically treated teeth restored with different post core systems—an in-vitro study. *Journal of Advanced Prosthodontics*.

[4] Akkayan B., Gülmez T. (2002). Resistance to fracture of endodontically treated teeth restored with different post systems. *Journal of Prosthetic Dentistry*.

[5] Papalexopoulos D., Samartzi T. K., Sarafianou A. (2021). A thorough analysis of the endocrown restoration: a literature review. *Journal of Contemporary Dental Practice*.

[6] Lovdahl P. E., Nicholls J. I. (1977). Pin-retained amalgam cores vs. cast-gold dowel-cores. *The Journal of Prosthetic Dentistry*.

[7] Sarabi N., Taji H., Jalayer J., Ghaffari N., Forghani M. (2015). Fracture resistance and failure mode of endodontically treated premolars restored with different adhesive restorations. *Journal of Dental Materials and Techniques*.

[8] Torabzadeh H., Ghassemi A., Sanei M., Razmavar S., Sheikh-Al-Eslamian S. M. (2014). The influence of composite thickness with or without fibers on fracture resistance of direct restorations in endodontically treated teeth. *Iranian Endodontic Journal*.

[9] Moosavi H., Ahrari F., Nojoomian M. (2010). Clinical evaluation of bonded amalgam restorations in endodontically treated premolar teeth: a one-year evaluation. *Journal of Contemporary Dental Practice*.

[10] Soares C., Silva N., Castro C. (2009). Influence of different post design and composition on stress distribution in maxillary central incisor: finite element analysis. *Indian Journal of Dental Research*.

[11] Asmussen E., Peutzfeldt A., Sahafi A. (2005). Finite element analysis of stresses in endodontically treated, dowel-restored teeth. *Journal of Prosthetic Dentistry*.

[12] Ni C.-W., Chang C.-H., Chen T. Y.-F., Chuang S.-F. (2011). A multiparametric evaluation of post-restored teeth with simulated bone loss. *Journal of the Mechanical Behavior of Biomedical Materials*.

[13] Pegoretti A., Fambri L., Zappini G., Bianchetti M. (2002). Finite element analysis of a glass fibre reinforced composite endodontic post. *Biomaterials*.

[14] Bilgin M. S., Erdem A., Dilber E., Ersoy İ (2016). Comparison of fracture resistance between cast, CAD/CAM milling, and direct metal laser sintering metal post systems. *Journal of Prosthodontic Research*.

[15] Fu G., Deng F., Wang L., Ren A. (2010). The three‐dimension finite element analysis of stress in posterior tooth residual root restored with postcore crown. *Dental Traumatology*.

[16] https://aalbadent.com/products/partial-denture-framework-alloys/vera-pdn-hard-reg.

[17] https://aalbadent.com/products/crown-bridge-alloys-fmc/npg.

[18] https://www.amanngirrbach.com/en/products/cadcam-material/metal/ceramill-cocr/.

[19] Kanduti D., Korat L., Kosec T., Legat A., Ovsenik M., Kopač I. (2021). Comparison between accuracy of posts fabricated using a digital CAD/CAM technique and a conventional direct technique. *International Journal of Prosthodontics*.

[20] Rodrigues S. A., Presotto A. G. C., Barão V. A. R., Consani R. L. X., Nóbilo M. A. A., Mesquita M. F. (2017). The roleof welding techniques in the biomechanical behavior of implant-supported prostheses. *Materials Science and Engineering: C*.

[21] Padrós R., Giner L., Herrero-Climent M., Falcao-Costa C., Ríos-Santos J. V., Gil F. J. (2020). Influence of the CAD-CAM systems on the marginal accuracy and mechanical properties of dental restorations. *International Journal of Environmental Research and Public Health*.

[22] Eid R., Juloski J., Ounsi H., Silwaidi M., Ferrari M., Salameh Z. (2019). Fracture resistance and failure pattern of endodontically treated teeth restored with computer-aided design/computer-aided manufacturing post and cores: a pilot study. *Journal of Contemporary Dental Practice*.

[23] Pang J., Feng C., Zhu X. (2019). Fracture behaviors of maxillary central incisors with flared root canals restored with CAD/CAM integrated glass fiber post-and-core. *Dental Materials Journal*.

[24] Aquilino S. A., Caplan D. J. (2002). Relationship between crown placement and the survival of endodontically treated teeth. *The Journal of Prosthetic Dentistry*.

[25] Salvi G. E., Siegrist Guldener B. E., Amstad T., Joss A., Lang N. P. (2007). Clinical evaluation of root filled teeth restored with or without post-and-core systems in a specialist practice setting. *International Endodontic Journal*.

[26] Rosenstiel S. F., Land M., Fujimoto J., Cockerill J. (2001). *Contemporary Fixed Prosthodontics*.

[27] Schwartz R. S., Robbins J. W. (2004). Post placement and restoration of endodontically treated teeth: a literature review. *Journal of Endodontics*.

[28] Sary S., Samah M. S., Walid A. A. Z. (2019). Effect of restoration technique on resistance to fracture of endodontically treated anterior teeth with flared root canals. *Journal of Biomedical Research*.

[29] Shah N., Makati D., Brave D., Singh Rathore V., Bhadra D., Dedania M. (2018). Evaluation of remaining dentin thickness and fracture resistance of conventional and conservative access and biomechanical preparation in molars using cone-beam computed tomography: an in vitro study. *Journal of Conservative Dentistry*.

[30] Mayya A., Naik R., Mayya S. S., Paul M. P. (2020). Fracture resistance of endodontically treated maxillary premolars with a longer single post and shorter double posts of different sizes: an in vitro study. *Journal of International Society of Preventive and Community Dentistry*.

[31] Khaledi A. A. R., Sheykhian S., Khodaei A. (2015). Evaluation of retention of two different cast post-core systems and fracture resistance of the restored teeth. *Journal of Dentistry*.

[32] Alharbi N., Wismeijer D., Osman R. B. (2017). Additive manufacturing techniques in prosthodontics: where do we currently stand? a critical review. *International Journal of Prosthodontics*.

[33] Ramos Júnior S., Felizardo K. R., Guiraldo R. D. (2021). CAD-CAM endodontic posts: literature review. *Research, Society and Development*.

[34] Pontius O., Hutter J., Giordano R., Schilder H., Hutter J. W. (2002). Survival rate and fracture strength of incisors restored with different post and core systems and endodontically treated incisors without coronoradicular reinforcement. *Journal of Endodontics*.

[35] Creugers N. H., Mentink A. G., Käyser A. F. (1993). An analysis of durability data on post and core restorations. *Journal of Dentistry*.

[36] Kantor M. E., Pines M. S. (1977). A comparative study of restorative techniques for pulpless teeth. *Journal of Prosthetic Dentistry*.

[37] Haghighi Z. B., Jahromy A. M. P. F. (2014). Comparison of fracture strength of endodontically treated teeth restored with two different cast metallic post systems. *Journal of Dental Biomaterial*.

[38] Gholami F., Kohani P., Aalaei S. (2017). Effect of nickel-chromium and non-precious gold color alloy cast posts on fracture resistance of endodontically treated teeth. *Iranian Endodontic Journal*.

[39] Khiavi H. A., Habibzadeh S., Safaeian S., Eftekhar M. (2018). Fracture strength of endodontically treated maxillary central incisors restored with nickel chromium and nonprecious gold alloy casting post and cores. *Journal of Contemporary Dental Practice*.

[40] Christian G. W., Button G. L., Moon P. C., England M. C., Douglas H. B. (1981). Post core restoration in endodontically treated posterior teeth. *Journal of Endodontics*.

[41] Hansen P. A., LeBlanc M., Cook N. B., Williams K. (2009). The quality of post and cores made using a reduce-time casting technique. *Operative Dentistry*.

[42] Sadeghi M. (2006). A comparison of the fracture resistance of endodontically treated teeth using three different postsystems. *Journal of Dental Medicine*.

